# Population genomics reveals multi-scale mechanisms sustaining schistosomiasis re-emergence in a near-elimination setting

**DOI:** 10.1371/journal.pntd.0014202

**Published:** 2026-09-08

**Authors:** Hannah D. Guss, Yannick Z. Francioli, Elise N. Grover, Andrew Hill, Wei Zou, Kristen J. Wade, Hamish Pike, Siddharth S. Gopalan, Liu Yang, Zhong Bo, David D. Pollock, Elizabeth J. Carlton, Todd A. Castoe

**Affiliations:** 1 Department of Biology, University of Texas at Arlington, Arlington, Texas, United States of America; 2 Department of Environmental and Occupational Health, Colorado School of Public Health, University of Colorado Anschutz, Aurora, Colorado, United States of America; 3 Department of Neurology, Weill Institute for Neurosciences, University of California San Francisco, San Francisco, California, United States of America; 4 Department of Molecular Genetics, Weizmann Institute of Science, Rehovot, Israel; 5 Sichuan Center for Disease Control and Prevention, Chengdu, The People’s Republic of China; 6 Department of Biochemistry & Molecular Genetics, University of Colorado School of Medicine, Aurora, Colorado, United States of America; IRCCS Sacro Cuore Don Calabria Hospital, ITALY

## Abstract

In China, sustained snail control, environmental management, and mass drug administration with praziquantel reduced schistosomiasis to near-elimination levels, yet re-emergence in Sichuan Province during the early 2000s exposed vulnerabilities in elimination efforts. We used population genomics to investigate the multi-scale population processes underlying *Schistosoma japonicum* re-emergence in Sichuan. We sequenced whole genomes from 270 miracidia collected from 53 human hosts across 17 villages in 2007, one year after re-emergence was documented. Population genomic analyses identified a broadly cohesive regional schistosome population with weak geographic structuring. Genome-wide diversity remained substantial, and demographic reconstructions revealed no recent decline in effective population size, suggesting that parasite populations had not undergone regional demographic collapse prior to re-emergence and were likely maintained in non-human reservoir hosts. At finer spatial scales, several villages exhibited reduced genomic diversity and elevated inbreeding, consistent with localized transmission maintained by relatively small founding populations. Estimates of pairwise genetic relatedness revealed dense within-village sibling clusters alongside second- and third-degree relationships spanning villages, and rare first- and second-degree cross-village links, supporting predominantly local transmission embedded within a connected regional transmission network. Genomic inference of minimum reproducing worm pairs identified substantial heterogeneity in host-level worm burden, ranging from one to eleven adult worm pairs, although uneven sampling limited absolute estimates. Together, these findings indicate that parasite persistence in this near-elimination setting was sustained by interacting processes operating across multiple biological scales, including diverse regional parasite populations, localized transmission networks connecting villages, and marked heterogeneity in host-level worm burden. More broadly, this work demonstrates how population genomics can reconstruct otherwise hidden patterns of parasite persistence and transmission, providing a valuable complement to conventional epidemiological surveillance in complex, multi-host parasite systems.

## Author summary

Schistosomiasis is a parasitic disease that affects millions of people worldwide. Understanding the factors that sustain parasite persistence and transmission, even in the face of control programs, is central to reducing and ultimately eliminating the disease. In China, decades of intensive control efforts have dramatically reduced infections, yet schistosomiasis persisted and re-emerged in some regions. In this study, we analyzed the genomes of 270 *S. japonicum* larvae collected from infected humans across 17 villages in Sichuan Province, China, shortly after schistosomiasis re-emerged in the region. Using population genomic analyses, we reconstructed the population processes underlying parasite persistence and transmission across regional, village, and host scales. Our findings suggest that, despite major reductions in human infection prevalence, schistosome population sizes do not show signatures of recent population decline, indicating that non-human hosts likely contributed to schistosomiasis persistence. Genetic relatedness among parasites also revealed that transmission remained highly localized, with relatively few infected snail hosts contributing disproportionately to infections within villages. Collectively, these findings suggest that parasite persistence reflected interacting processes operating across multiple biological scales, including non-human reservoirs, regional connectivity, localized transmission networks, and heterogeneous host-level worm burdens, highlighting challenges for elimination and demonstrating the value of population genomics for genomic surveillance.

## Introduction

Schistosomiasis is a neglected tropical disease that has been targeted for elimination as a public health problem by the World Health Organization [1], but the re-emergence and persistence of infections in transmission hotspots are a key barrier to attaining that goal [2,3]. Schistosomiasis is caused by parasitic blood flukes of the genus *Schistosoma* that affects more than 250 million people worldwide and contributes substantially to chronic morbidity, including hepatic fibrosis and anemia [4,5]. *S. japonicum*, which inhabits much of mainland China as well as the islands of Indonesia and the Philippines, has a complex life cycle involving mammalian definitive hosts and *Oncomelania* snails as obligate intermediate hosts. Eggs shed in mammalian feces hatch into miracidia that infect snails, where they undergo asexual clonal amplification before releasing cercariae that infect mammalian hosts through the skin and mature into adult worms within the hepatic portal vasculature [5,6]. Among human schistosomes, *S. japonicum* is distinguished by its broad mammalian host range and zoonotic transmission ecology, infecting humans as well as bovines and other domestic and wild mammals [7–9]. Over the past seven decades, China has implemented one of the most sustained and comprehensive schistosomiasis control programs globally, combining large-scale snail control, environmental modification, health education, and mass drug administration (MDA) with praziquantel (PZQ) [10–12]. These coordinated interventions substantially reduced prevalence in many regions and brought transmission to near-elimination levels [2,13]. From both epidemiological and theoretical perspectives, such sustained reductions in prevalence are expected to push parasite populations toward transmission breakpoints and eventual collapse.

Classical macroparasite transmission theory predicts that sustained reductions in worm burden decrease mating probability and can ultimately drive parasite populations below density-dependent transmission breakpoints [14–16]. Because schistosomes are sexually reproducing and require male-female pairing within hosts, reductions in worm density can generate nonlinear declines in reproductive success. Under such models, declining human prevalence that goes below approximately 1% is expected to approach thresholds at which transmission becomes unsustainable in the absence of continued reintroduction via spatial connectivity or alternative host reservoirs [15]. Yet in the early 2000s, schistosomiasis re-emerged in mountainous regions of Sichuan Province despite prevalence having previously been estimated to be below this threshold [2]. By 2004, renewed local transmission had been confirmed in seven of the 21 Sichuan counties that had achieved transmission-control status and in one of the 25 counties that had achieved transmission-interruption status [2,17]. Yet this resurgence occurred despite persistently low infection intensities, which averaged only 1.6 eggs per gram (EPG) of stool across the region in 2007 and reached a maximum village-level mean of just 10.6 EPG (S1 Table; [17]).

Several non-mutually exclusive mechanisms could explain the persistence and/or re-emergence of transmission near elimination. First, effective population size (N_e_) may remain sufficiently large to buffer demographic collapse despite low observed human prevalence, particularly if *S. japonicum* populations are harbored across multiple reservoir host species. In multi-host systems, reductions in human prevalence may not translate directly into proportional reductions in schistosome population size if non-human reservoirs maintain transmission cycles [18–20]. Second, focal transmission may persist locally due to the survival of intermediate snail hosts or pockets of viable snail habitat that permit small founder populations to expand [8,11]. Third, regional connectivity via hydrological networks or host movement may prevent epidemiological fragmentation, allowing *S. japonicum* lineages to reseed local populations even after substantial reductions in prevalence [21–23].

Disentangling these mechanisms requires approaches that integrate demographic, spatial, and within-host scales of inference. Population genomics provides such an approach by enabling reconstruction of effective population size trajectories, quantification of genetic connectivity, and identification of close kin relationships among parasites [20,24–26]. Because the cercarial life stage can be difficult to find and sample effectively, assessing schistosome populations through whole-genome sequencing of individual miracidia from fecal material provides a powerful non-invasive sampling approach. These genomic data can reveal otherwise hidden population processes underlying persistence, connectivity, and transmission that are not apparent from conventional epidemiological measures such as prevalence alone.

Here, we leverage whole-genome sequencing of 270 *S. japonicum* miracidia collected from 53 human hosts across 17 villages in Sichuan Province in 2007, the year following documented re-emergence. We test key assumptions of density-dependent breakpoint models by evaluating whether sustained reductions in human prevalence were accompanied by detectable genomic evidence of *S. japonicum* population decline, whether schistosome populations became spatially fragmented consistent with disruption of local transmission networks, whether regional connectivity persisted at levels sufficient to reseed local populations, and whether within-host schistosome structure revealed heterogeneity in worm burden that could enable a subset of hosts to disproportionately sustain transmission. By integrating genomic demographic inference with fine-scale relatedness analyses, we assess whether re-emergence reflects demographic collapse and episodic reseeding or instead sustained multi-scale transmission processes in which reductions in human prevalence did not translate into collapse of *S. japonicum* populations.

## Materials and methods

### Ethics statement

This study was conducted in accordance with the Declaration of Helsinki. All sampling procedures were approved by the Sichuan Institutional Review Board, the University of California, Berkeley Committee for the Protection of Human Subjects, and the Colorado Multiple Institutional Review Board (protocol 15–1059). Written informed consent was obtained from all participants. Individuals who tested positive for *S. japonicum* infection were notified and referred to the local anti-schistosomiasis control station for treatment according to standard protocols.

### Sample collection

270 *Schistosoma japonicum* miracidia were collected in 2007 from 53 human hosts across 17 villages in three counties of Sichuan Province, China, during village-wide infection surveys conducted as previously described [17]. Participants were screened for infection using the miracidia hatching test using stool samples collected over up to three consecutive days. Miracidia emerging from positive hatching assays were individually isolated using a hematocrit tube or flame-drawn Pasteur pipette, washed three times in autoclaved deionized water, and transferred onto Whatman FTA classic indicating cards (GE Healthcare) for long-term storage [27]. Human sampling and informed consent procedures are described in the Ethics Statement section below. From the archived collection, we selected multiple miracidia per infected host across villages spanning approximately 75 km to maximize representation of regional genetic diversity. Not all infected hosts or villages are represented in the final dataset due to whole-genome amplification or sequencing failures, and because a subset of samples had been previously used for reduced-representation sequencing [28].

### Whole genome amplification and sequencing

Miracidia were recovered from Whatman FTA classic cards using a Whatman Harris 2 mm micro-core punch (Whatman; cat. WB100029). Whole genome amplification (WGA) was performed directly from card punches following previously described protocols [24,28] using the Illustra Ready-To-Go GenomiPhi V3 DNA Amplification Kit (GE Healthcare; cat. 25-6601-96). Detailed methods for WGA, library preparation and sequencing are provided as supporting information (S1 and S2 text). Amplified DNA quantity was assessed using a Qubit fluorometer, and samples yielding >20 ng were selected for whole-genome shotgun library preparation. Sequencing libraries were prepared from individual amplified samples using Illumina Nextera Flex kits, pooled in multiplexed sets of up to 96 libraries, and sequenced on an Illumina NovaSeq 6000 S4 flow cell with 150 bp paired-end reads. Sequencing depth targeted approximately 20 × genome coverage per miracidium. A total of 388 archival miracidial samples were whole genome amplified, of which 315 produced >5 ng of DNA and proceeded to library preparation and sequencing. Of these, 41 failed to meet the minimum thresholds of 20x sequencing coverage and 85% raw reads mapping to the reference. An additional four were excluded because of excessive missing genotype data, leaving 270 samples used for downstream analyses.

### Read processing and variant identification

Across the 270 samples, the mean paired-end sequencing reads per sample was 75 million, the mean depth of coverage was 37x, and the mean percentage of reads that mapped to the reference *S. japonicum* genome [29] was 95% (S1A and S1B Fig). Because whole-genome amplification is non-selective and amplifies all DNA present on the FTA cards, the proportion of raw sequencing reads mapping to the S. japonicum reference genome was used as a quality-control metric, serving as a proxy for parasite DNA content while also identifying potential host or environmental contamination. We quality filtered raw reads using Trimmomatic v.0.39 with the following options: LEADING:20 TRAILING:20 MINLEN:75 AVGQUAL:20 and then mapped our trimmed and quality filtered reads to the *S. japonicum* reference genome (SjV3; ASM636876v1; [29]) using default parameters in BWA. The SjV3 assembly was generated from a single clone of male worms of Chinese mainland origin, comprises 408.5 Mb anchored across eight pseudo-chromosomes with a scaffold N50 of 48.8 Mb and a quality value (QV) score of 37 [29]. We called variants using GATK v.4.0.8.1 using the best practices workflow [30] by first generating individual variant call files (VCFs) using ‘HaplotypeCaller’, specifying –ERC GVCF and then called population variants using ‘GenotypeGVCFs’. We then used GATK ‘VariantFiltration’ to further hard filter based on GATK’s best practices recommendation (QD < 2, QUAL < 30.0, SOR > 3.0, FS > 60.0, MQ < 40.0, MQRankSum < -12.5, ReadPosRankSum < -8.0, cluster-size 3, cluster-window-size 10) and then used the module ‘SelectVariants’ to exclude non-filtered variants. We used VCFtools [31] to remove indels and retain biallelic sites that had a MAF > 0.05, genotype quality > 30 and removed sites that were missing more than 80% of genotype calls. After filtering variants for quality, completeness, and masking annotated genomic repeat elements, the final VCF contained 12,710,408 high quality single nucleotide polymorphisms (SNPs). For analyses sensitive to linkage disequilibrium or data density, we generated a reduced dataset by randomly selecting a single SNP per 10kb window, yielding 54,890 SNPs (hereafter referred to as the 10kb-thinned dataset). Because of the high proportion of repetitive DNA found in the *S*. *japonicum* genome (~45%), and the difficulty in accurately calling variants in repeat regions, we also masked repetitive elements using the repeat annotation file from the *S. japonicum* reference genome (ASM636876v1) and removed all variants found on the Z chromosome.

### Population clustering and structure

To examine the distribution of genetic variance, we performed a principal components analysis (PCA) in PLINK v1.9 using our full SNP dataset. We used R version 4.4.3 to calculate the percent of variance explained by each principal component. We used the program ADMIXTURE v.1.3 [32] to estimate population structure using only our 10kb-thinned dataset. We used the cross-validation method implemented in ADMIXTURE to determine the best fit value of *K* from a range of values (*K =* 2–10). Using the same 10kb-thinned dataset, we constructed a rooted neighbor-joining phylogeny using pairwise p-distances computed across thinned autosomal SNPs using PLINK v1.9. The resulting distance matrix was used to infer a neighbor-joining tree with the nj() function in the R package ape [33]. The tree was rooted using two geographically distinct *S. japonicum* outgroup samples (SRR17253232, Indonesia; SRR17253272, Yunnan Province, China [29]). Node support was assessed via non-parametric bootstrapping: 10,000 pseudo-replicate SNP matrices were generated by resampling the thinned SNP set with replacement, and a neighbor-joining tree was rebuilt from each replicate’s pairwise distance matrix, with node support reflecting the percentage of replicates recovering each clade in the original tree. The rooted tree is presented in the main text without bootstrap collapsing; a version with nodes below 85% bootstrap support collapsed into polytomies is provided in the supplement. Both trees were plotted in R using ggtree, with tips colored according to village identity to facilitate interpretation of spatial population structure.

### Estimating genomic diversity

To quantify genomic diversity across samples and villages, we estimated per-sample heterozygosity, genome-wide inbreeding coefficients (F), and nucleotide diversity (π) from the quality-filtered SNP dataset. Heterozygosity and inbreeding coefficients were calculated from individual-level genotype counts using the PLINK --het framework, with heterozygosity estimated as 1−(OHOM/N_SITES). We summarized heterozygosity and F values for each miracidium and compared their distributions across villages. To estimate nucleotide diversity, we calculated genome-wide π in sliding windows and generated per-sample π estimates for comparison with individual inbreeding coefficients. We then used R version 4.4.3 to visualize variation in heterozygosity, F, and π across villages and geographic groups, and to test the relationships between F and both heterozygosity and per-sample π using linear models.

### Demographic inferences

To infer the demographic history of the Sichuan *S. japonicum* population, we first used easySFS [34] to generate a folded site frequency spectrum (SFS), with projection set to 540 to retain all sites across individuals, resulting in 6,157,643 segregating sites. We then ran Stairway Plot v2.1.1 [34] using the assembled genome size (344 Mb) as the sequence length, 540 chromosomes, and assuming a generation time of 0.5 years and a mutation rate of 8.9 × 10^−9^ per site per generation, consistent with published estimates for schistosomes [26]. We used default Stairway Plot parameters, including 200 bootstrap replicates and training on 67% of sites per replicate to obtain confidence intervals around the inferred demographic history. To further validate demographic inferences, we also applied SMC++ [35] using the same generation time and mutation rate parameters described above. We estimated population history over the interval of 10–100,000 generations, including all 270 samples and designating three as distinguished individuals. Variant data were split by chromosome with VCFtools [31] and converted to SMC input format for each distinguished individual using the vcf2smc command, with runs of homozygosity longer than 50 kb masked as missing. Demographic inference was then performed with the estimate command across all input files. To evaluate uncertainty, we conducted bootstrap analyses by resampling 10 randomly selected 10 Mb segments per chromosome.

To evaluate the sensitivity of our demographic inference approaches to a potential recent bottleneck, we performed coalescent simulations using msprime v1.4.2 [36]. Four demographic scenarios were simulated: a constant-size control (Ne = 55,046, matching the ancestral Ne inferred from the empirical data) and three bottleneck scenarios in which N_e_ declined 2-fold, 10-fold, or 50-fold beginning 60 generations before sampling, corresponding to the onset of mass drug administration in 1977 under an assumed generation time of 0.5 years. Each scenario was simulated across seven 5-Mb chromosomes (35 Mb total) for 270 diploid individuals under a mutation rate of 8.9 × 10^−9^ and recombination rate estimated from *S. mansoni* of 3.4 × 10^−8^ per site per generation [37], with 20 replicates per scenario. Simulations were analyzed with both Stairway Plot (200 bootstrap replicates per simulation replicate) and SMC++ (60 subsampled individuals, 5 simulation replicates lineages), but with a reduced set of simulated data for SMC++ required to accommodate the higher computational intensity of SMC++ inference.

### Inferences of fine-scale relatedness

To assess patterns of genetic relatedness across the *S. japonicum* population, we used the RAB (Ratio of Allele Balance) metric implemented in NGSrelate v2 [38]. This method leverages genotype likelihoods to estimate pairwise relatedness and is particularly well suited for populations with high inbreeding. In this framework, RAB represents the probability that two individuals share alleles identical by descent (IBD) at a given locus accounting for inbreeding (Hedrick and Lacy 2015). We also used the Rare Allele sharing (RAS) pipeline [39,40], to infer RAS values between pairs of miracids. For both of these inferences, we first pruned the dataset using MAF >= 0.1 and missingness <= 0.2 filters, and pruned SNPs in strong linkage disequilibrium using PLINK v2.0 [41] by scanning the genome in sliding windows of 50 SNPs advancing by 10 SNPs and removing variants with pairwise r^2^ > 0.2 within each window. We visualized pairwise RAB values as heatmaps in R using ggplot2, with samples ordered by village in a predefined north-to-south sequence and, within villages, by host identity. Heatmap cells were colored according to either binned or continuous RAB values, and village boundaries were annotated along both axes to facilitate interpretation of within-host, within-village, and between-village patterns of relatedness.

Both RAB and RAS were further benchmarked against pedigree estimates reconstructed with the R package Sequoia v3.0.3 [42], which infers parent–offspring and sibship relationships via a maximum-likelihood framework and COLONY [43], another pedigree inference software. Analyses in Sequoia and COLONY were conducted using the original LD-pruned dataset used for RAS and RAB inferences, which was then further 100-kb–thinned (2,962 SNPs). This filtering approach followed recommendations in the documentation of these programs, and is required to accommodate the computational burden of pedigree reconstruction. We identified candidate related miracid pairs using the Sequoia command “GetMaybeRel”, the “ped” module with CalcLLR = TRUE, a conservative genotype error rate (Err = 0.01), discrete generations, and summarized log-likelihood ratios (LLR) and assignment confidence (EstConf). For an independent cross-check, we inferred sibships with COLONY, treating loci as diploid/dioecious, allowing polygamy for both sexes and inbreeding, and assigning conservative genotyping error rates (allelic drop-out and other error = 0.01).

To convert continuous RAB values into categorical relatedness classes, we constructed empirical training sets for 1^st^ degree and unrelated (defined as 4th+ degree) relationships and then fit a simple normal-mixture framework to generate posteriors of relatedness degrees for every miracid pair. First, we defined a high-confidence full-sib set by intersecting Sequoia full-sib calls with COLONY full-sib dyads. We then defined an “unrelated” set by selecting pairs classified as unrelated by both methods and sampled from geographically distant villages (pairs between northern and southern groups). From these two reference sets, we estimated the empirical mean and variance of RAB for full siblings and unrelated pairs. Expected RAB means ($\hat{\mu }$_degree_) and variance ($\hat{\sigma }$_degree_) for intermediate degrees of relatedness were then approximated by placing successive means halfway between the sibling and unrelated expectations, and by reducing the sibling variance by half for each additional degree of separation, reflecting the increased number of meiosis events (${\hat{\mu }}_{\text{2}\circ }=\frac{\left(\left(\;{\hat{\mu }}_{unrelated}+\;{\hat{\mu }}_{sibs}\right)\right)}{\text{2}}$ and ${\hat{\sigma }}_{\text{2}\circ }^{\text{2}}=\frac{{\hat{\sigma }}_{sibs}^{\text{2}}}{\text{2}}$). These parameters defined a four-component normal mixture corresponding to 1st-, 2nd-, 3rd-degree, and unrelated. Assuming equal prior probabilities across classes, we computed posterior probabilities for each category for every observed pairwise RAB value. For downstream analyses, we retained and summarized pairs whose maximum a posteriori (MAP) classification exceeded 0.95 posterior probability. Based on this posterior calibration, RAB thresholds corresponding to ≥95% posterior probability were defined as follows: first-degree (full sibling or parent–offspring) relationships were assigned at RAB > 0.425; second-degree relationships corresponded to RAB values between 0.231 and 0.425; third-degree relationships were defined as RAB values between 0.0166 and 0.231; and values < 0.0166 were classified as fourth-degree or unrelated. These calibrated thresholds were used consistently in downstream host-, village-, and network-level analyses, as well as in figure visualizations where RAB cutoffs are applied.

To estimate the minimum number of distinct reproducing worm pairs per host, we counted the number of unique full-sibling clusters (RAB > 0.425) identified within each host, with each sib cluster interpreted as the minimum evidence for one unique reproducing adult worm pair. To quantify aggregation in inferred worm burden across hosts [44], we fit a zero-truncated negative binomial distribution to sibling cluster counts across all 53 hosts using the posnegbinomial() family in VGAM [45], which is appropriate because every host in our dataset yielded at least one sampled miracidium, making true zero counts unobservable.

To visualize relatedness patterns, we generated network and circular (circos) representations from pairs classified with ≥0.95 posterior probability. Full-sibling clusters within hosts were defined using the calibrated first-degree RAB threshold (RAB > 0.425), and for cluster-level visualizations, multiple dyadic links between the same pair of clusters were collapsed to a single connection, retaining the highest relatedness class observed. Network plots were constructed in R v4.4.3 using the packages *igraph* and *ggraph*, applying a Fruchterman–Reingold force-directed layout. Circos visualizations were generated using the *circlize* package, with villages ordered north–south; host-level tiles represent individual hosts and cluster-level tiles represent inferred sibling clusters, with stacked points (and tile height in cluster panels) reflecting the number of sequenced miracidia. Links were drawn between hosts or clusters, colored by relatedness degree and scaled by the number of contributing dyads, and an outer track denotes geographic village groupings.

## Results

### Weak regional genetic structure supports cohesive demographic inference

**Fig 1:**
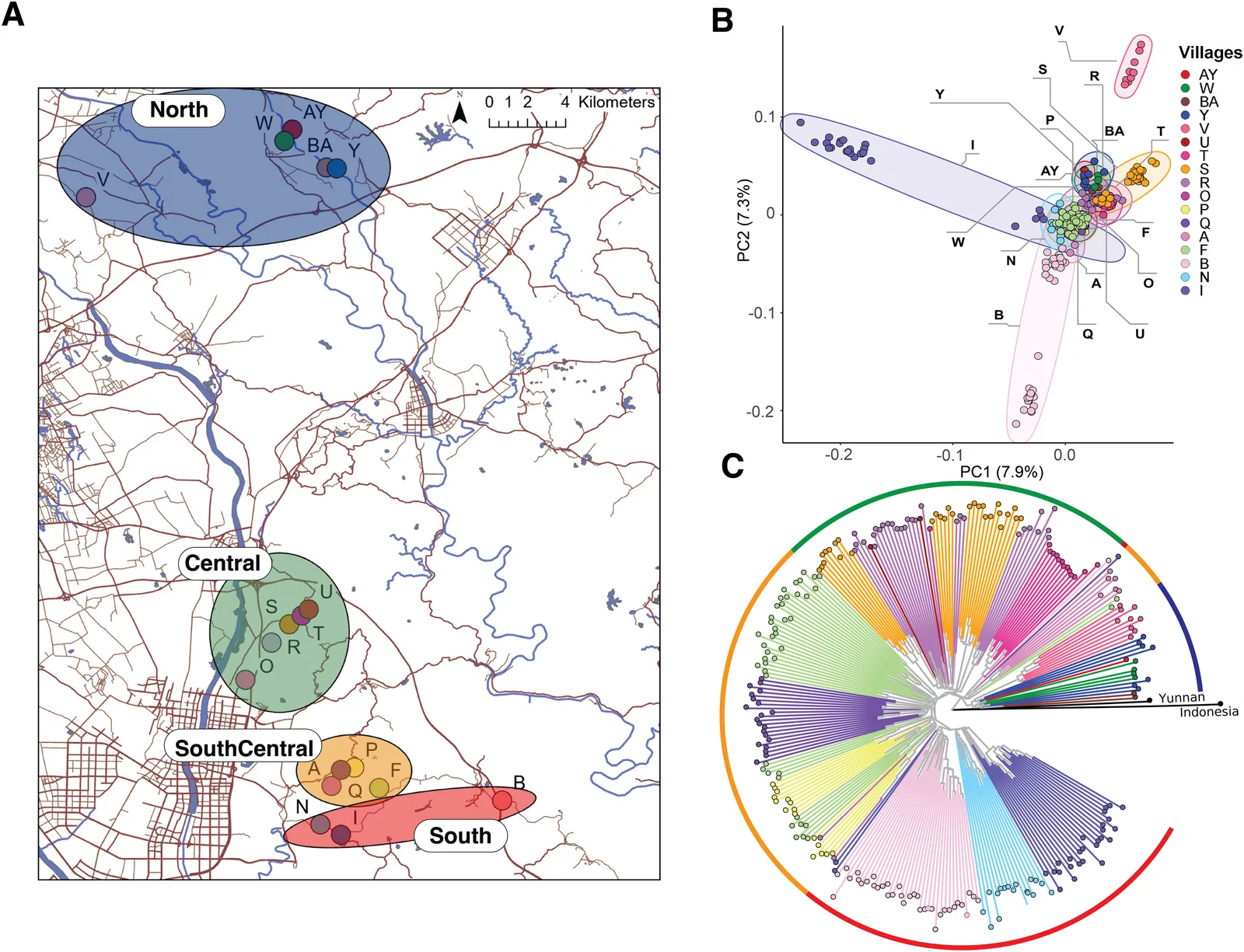
Geographic sampling and broad population genomic structure of Schistosoma japonicum miracidia in Sichuan Province, China. (A) Map showing the locations of 17 sampled villages. Colored dots denote villages, with major waterways (blue) and roads (brown) providing geographic context. Geographic grouping are indicated by colored ovals. This is an original map created in ArcGIS Pro 3.6, using open source resources and data made available by ESRI, TomTom, Garmin, FAO, NOAA, CGIAR, USGS, Geofabrik GmbH, OpenStreetMap Contributors and the GIS Community. The OpenStreetMap license information and ESRI-compatible shape files used to generate this map are available at: https://download.geofabrik.de/asia/china/sichuan.html (B) Principal component analysis of genome-wide SNP variation among 270 miracidia. Most samples form a single broadly overlapping cluster, with parasites from geographically peripheral northern and southern villages showing modest differentiation. (C) Neighbor-joining tree based on pairwise genetic distances from a 10-kb-thinned SNP dataset, rooted with outgroup samples from Yunnan Province, China, and Indonesia. Branches are colored by village, illustrating finer-scale clustering within villages and among geographically proximate villages. Colored arcs around the tips indicate the geographic groups shown in panel A. A version of the tree with branches receiving <85% bootstrap support collapsed is provided in S4 Fig.

The 270 whole genome amplified miracidia included in this analysis came from 53 human hosts and 17 villages dispersed across four geographic regions: North (N = 5), Central (N = 5), South Central (N = 4) and South (N = 3) (Fig 1A and S2 Table). PCA of the full SNP dataset (12,710,408 SNPs) revealed a largely cohesive regional population, with samples from most villages forming a single overlapping cluster along the first two principal components. Modest differentiation was observed only among villages at the northern and southern geographic periphery (Fig 1B). Samples from village V, a geographically distant northern village (Fig 1A), formed a distinct cluster, while a subset of samples from the southernmost villages B and I also separated along the same axes (Fig 1B). Analysis of our 10 kb–thinned SNP dataset using ADMIXTURE (excluding all closely related individuals (1^st^ and most 2^nd^ degree with RAB < 0.3; 54,890 SNPs) suggested K = 1 as the optimal number of genetic clusters, while K = 2 captured a weak north–south gradient (S2 Fig). Together, PCA and ADMIXTURE support the presence of a largely cohesive regional population with modest north–south differentiation.

Phylogenetic analysis of the 10-kb-thinned dataset revealed additional fine-scale genetic structure within and among villages that was not apparent from the PCA or ADMIXTURE analyses (Fig 1C). However, bootstrap support for many basal nodes connecting villages was low, limiting confidence in the precise relationships among villages. Consistent with this, collapsing branches with <85% bootstrap support (S3 Fig) also reduced much of the apparent mixing among villages while preserving the major patterns of village-level clustering. Despite this limited basal support, samples from individual villages generally clustered together, and geographically proximate villages tended to form regional groupings. For example, the southernmost villages (B, N, and I) largely clustered together, with the exception of a single miracidium from village B. Similarly, samples from the northernmost villages (AY, W, BA, and Y) exhibited nearly exclusive within-village clustering (Fig 1C). In contrast, samples from several central and south-central villages (e.g., S, F, P, and R) showed comparatively weaker village-specific clustering. Collectively, PCA, ADMIXTURE, and phylogenetic analyses support a largely cohesive regional population exhibiting weak geographic differentiation, with finer-scale genetic structure primarily evident within villages and among neighboring villages.

### Genomic diversity

**Fig 2:**
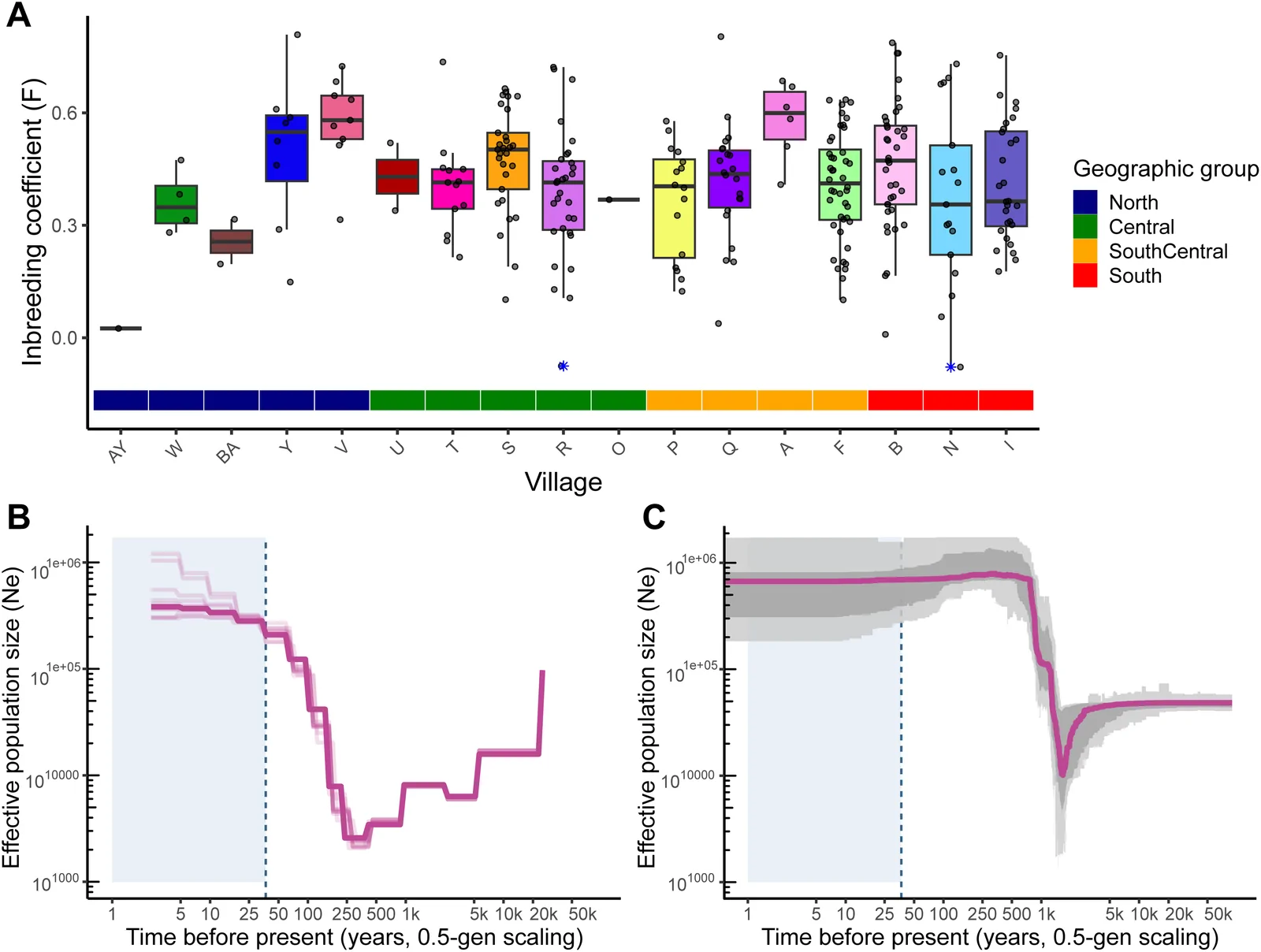
Genomic diversity, inbreeding, and historical demography of Schistosoma japonicum in Sichuan Province. (A) Genome-wide inbreeding coefficients ($\bar{F}$) for individual miracidia summarized by village. Points represent individuals and boxplots show village-level distributions, colored by geographic region (B) Historical effective population size (N_e_) inferred using the sequential Markov coalescent approach (SMC++). The inferred demographic trajectory shows a pronounced historical decline without evidence of a substantial recent reduction during the period of modern schistosomiasis control (blue dotted line and shaded region). (C) Historical effective population size (N_e_) inferred using Stairway Plot from the site frequency spectrum. The inferred trajectory similarly indicates a pronounced long-term population decline beginning approximately 1,500 years before present, followed by partial recovery and stabilization. Together, these analyses indicate no evidence of a substantial recent decline in S. japonicum effective population size despite documented reductions in human prevalence.

Given the history of intensive schistosomiasis control in this region, including repeated mass drug administration and improvements in sanitation, we expected S*. japonicum* populations to exhibit reduced genomic diversity and elevated inbreeding. Genome-wide inbreeding coefficients ($\bar{F}$) estimated for each miracidium varied widely among individuals ($\bar{F}$ = –0.16 to 0.74) and among villages (mean $\bar{F}$ values 0.025–0.579), indicating substantial heterogeneity in inbreeding levels (Fig 2A). Villages A ($\bar{F}$ = 0.58), V ($\bar{F}$ = 0.58), and Y ($\bar{F}$= 0.54) exhibited the highest $\bar{F}$ values, while villages R ($\bar{F}$ = 0.39), P ($\bar{F}$ = 0.40), and N ($\bar{F}$ = 0.42) had lower mean values and broader distributions, particularly in villages R and N (Fig 2A).

As expected, inbreeding coefficients ($\bar{F}$) were almost perfectly inversely correlated with individual heterozygosity (R² ≈ 1), reflecting the mathematical relationship between these metrics. More importantly, ($\bar{F}$) was also negatively correlated with village-level nucleotide diversity (π) (R² = 0.72, p < 0.05), indicating that villages with lower genomic diversity also tended to harbor more highly inbred parasites. Together, these patterns reveal pronounced spatial heterogeneity in genomic diversity, with some villages exhibiting elevated inbreeding and reduced diversity whereas others exhibited moderate to high genomic diversity. Notably, the persistence of moderate to high diversity across multiple villages is inconsistent with a uniform regional genetic bottleneck prior to re-emergence.

### No genomic evidence of recent demographic collapse

To further evaluate whether the heterogeneous patterns of genomic diversity observed across villages reflect a recent regional demographic contraction, rather than village-specific founder effects or spatially restricted transmission, we inferred historical changes in *S. japonicum* effective population size (N_e_) using two complementary genome-wide approaches: the site frequency spectrum–based Stairway Plot and the sequential Markov coalescent framework SMC++. Both analyses inferred a pronounced decline in N_e_ centered approximately 1,500 years ago, followed by partial recovery and subsequent stabilization (Fig 2B and 2C). This historical decline substantially predates modern schistosomiasis control efforts initiated in the 1970s [11] and likely reflects older environmental or sociopolitical changes, such as shifts in land use, climate, or human settlement.

Because coalescent-based N_e_ inference can exhibit reduced sensitivity or spurious signal at shallow time depths [46], we performed simple coalescent simulations in msprime as a sensitivity analysis to assess whether our analytical pipeline could detect recent demographic contractions comparable in timing to modern control efforts. These simulations necessarily assumed a simplified panmictic population history and did not incorporate important biological features of *S. japonicum*, including spatial population structure, metapopulation dynamics, or the complex multi-host life cycle. Using a constant-N_e_ null model and 2-, 10-, and 50-fold bottlenecks beginning 60 generations before sampling (approximating the onset of mass drug administration in 1977), simulated datasets were analyzed using the same Stairway Plot and SMC++ pipelines. Under these simplified demographic scenarios, Stairway Plot detected 100% of simulated 10-fold and 50-fold bottlenecks and 45% of 2-fold bottlenecks, whereas SMC++ detected 80% of 2-fold, 100% of 10-fold, and 60% of 50-fold bottlenecks (S4 Fig).

Taken together, these results suggest that the absence of a recent decline in inferred N_e_ is unlikely to reflect limited statistical power alone, although our simplified simulations do not capture the full demographic complexity of natural S. japonicum populations. Accordingly, we interpret the lack of genomic evidence for a recent demographic contraction as being consistent with no substantial decline in effective population size during the modern control period. Overall, despite well-documented declines in human prevalence, we found no genomic evidence of a recent regional demographic collapse in S. japonicum, highlighting a disconnect between human case counts and parasite effective population size.

### Fine-scale relatedness patterns reveal village-level genetic clustering

To characterize patterns of fine-scale relatedness across our sampling, we compared RAB and RAS IBD approaches, each benchmarked against pedigree-based reconstructions generated using Sequoia and Colony. Although RAS and RAB were positively correlated (R² = 0.388), RAB more accurately recapitulated pedigree-based expectations: full-sibling pairs independently identified by Sequoia and Colony clustered near the expected IBD value of ~0.5 under RAB, whereas RAS showed greater dispersion and inflation among unrelated pairs (S5A–S5C Fig). We therefore used RAB as the primary estimator of relatedness and calibrated posterior probability thresholds for relationship classes based on full-sibling pairs (from a single host) identified by both pedigree methods (mean RAB = 0.566; variance = 0.0031; n = 262 sibling pairs; see Methods).

**Fig 3:**
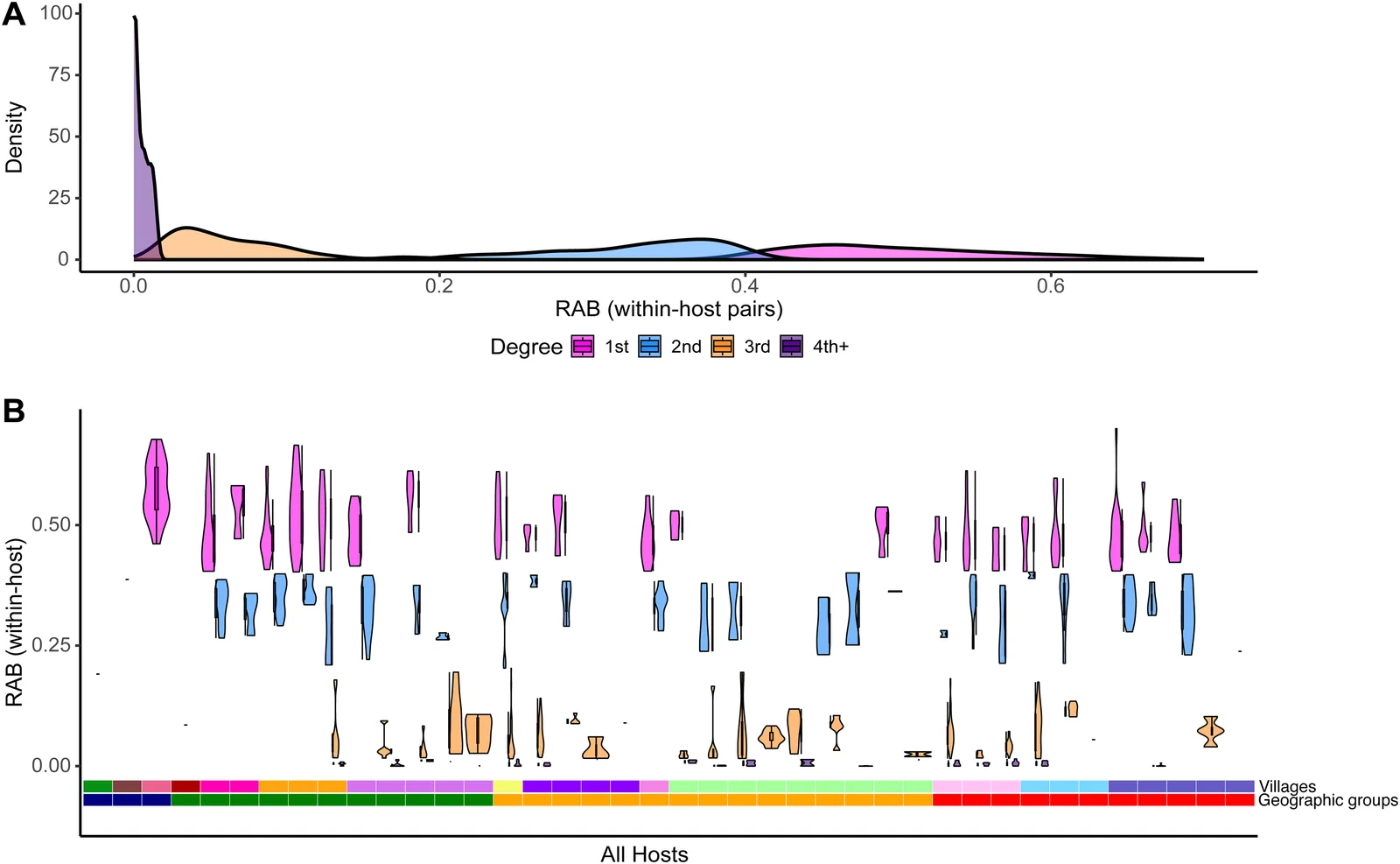
Within-host distribution of RAB-inferred relatedness among Schistosoma japonicum miracidia. (A) Density of Rare Allele Balance (RAB) values for miracidial pairs within hosts, colored by pedigree-calibrated relatedness class. (B) Host-specific distributions of within-host RAB values across all sampled hosts, with bars indicating village membership. Peaks corresponding to first- and second-degree relatedness are consistent with clonal infections arising from single infected snails, whereas elevated third-degree relatedness is consistent with localized inbreeding. Together, these patterns reveal extensive relatedness among miracidia within hosts and among parasites sampled from the same villages.

**Fig 4:**
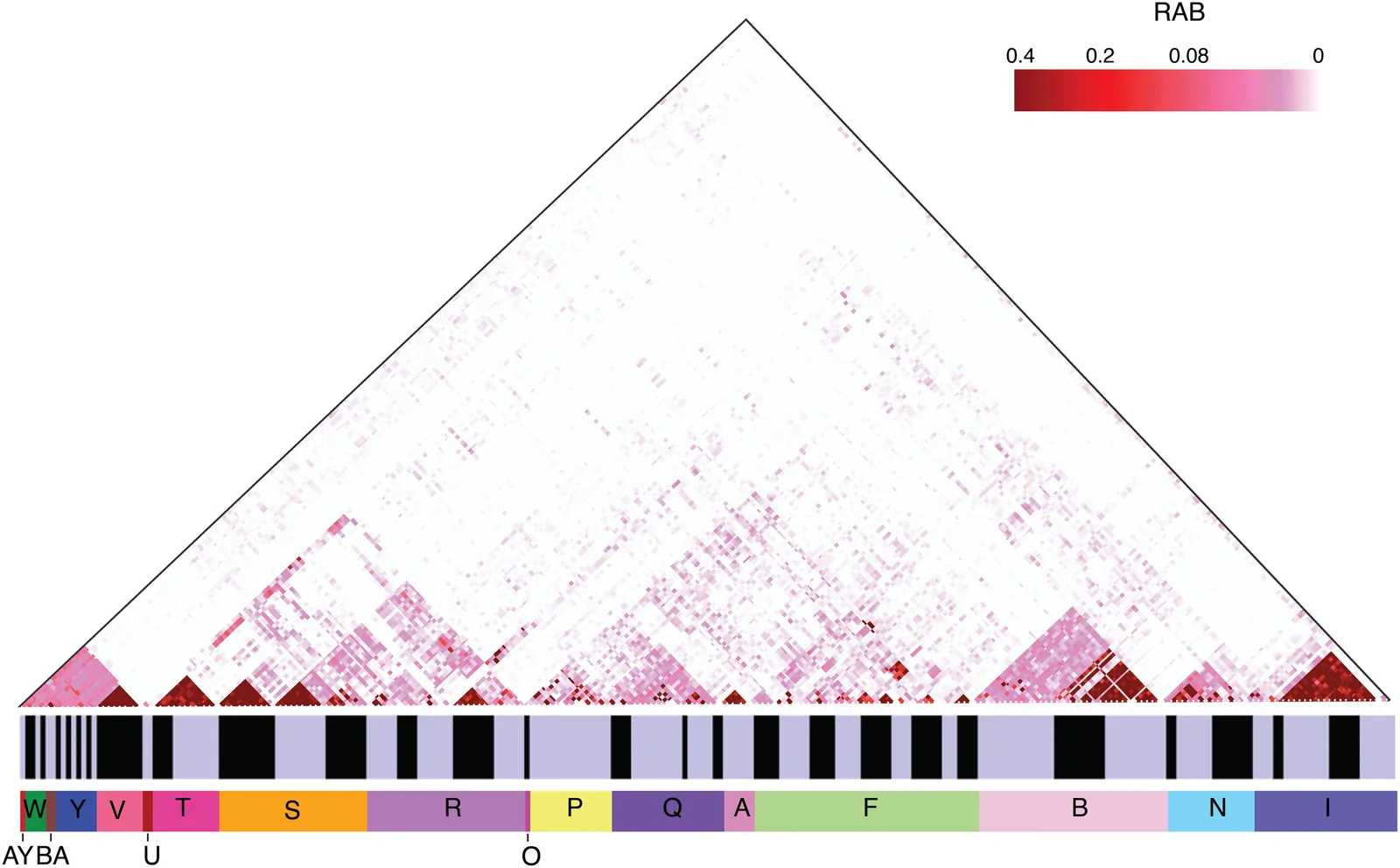
Genome-wide relatedness among Schistosoma japonicum miracidia. Triangle heatmap of pairwise allele-sharing (RAB) across all samples, ordered by village along a north–south gradient with hosts marked by alternating bars. High-RAB triangles (> 0.4) indicate within-host sibling groups, whereas broader regions of moderate relatedness (0.1–0.4) reflect relationships shared among hosts within villages and geographic clusters. Although relatedness shows hierarchical spatial structure, several first- and second-degree connections span distant villages (e.g., R, P, and U), indicating transmission links not explained solely by geographic distance.

Closely related parasites were common within individual hosts (n = 53): 70% of hosts harbored at least one first-degree (full-sibling) miracidial pair and 80% harbored at least one second-degree pair (Fig 3A and 3B). The frequent co-occurrence of half-siblings within hosts is consistent with repeated exposure to clonal cercariae originating from single infected snails, which produce genetically identical cercariae during asexual amplification. Considering the frequency of half-sib clusters, some full-sibling clusters may also represent clonal pairs. These within-host patterns are visually apparent in the RAB heatmap (Fig 4), where within-host sibling groups form distinct triangular blocks of high relatedness along the diagonal.

At the village scale, the heatmap reveals larger triangular blocks of moderate relatedness among hosts within the same village (Fig 4), including first- and second-degree relationships shared across individuals. We observed clusters of first- and second-degree related miracidia infecting different individuals within villages, consistent with shared exposure to the same infected snails or closely related parasite lineages circulating locally. In some villages (e.g., S and I), infections were composed almost exclusively of first- and second-degree relatives, producing prominent triangular relatedness blocks indicative of highly clustered local transmission. In contrast, other villages exhibited broader within-village relatedness distributions, consistent with contributions from a more genetically heterogeneous local parasite population.

Together, the within-host half-sibling relationships and within-village relatedness blocks indicate that transmission within villages is highly localized, with infections frequently involving closely related parasite lineages. The persistence of these dense local clusters despite low overall regional prevalence suggests that parasite transmission remains concentrated within localized transmission networks.

### Cross-village relatedness indicates limited but persistent regional connectivity

**Fig 5:**
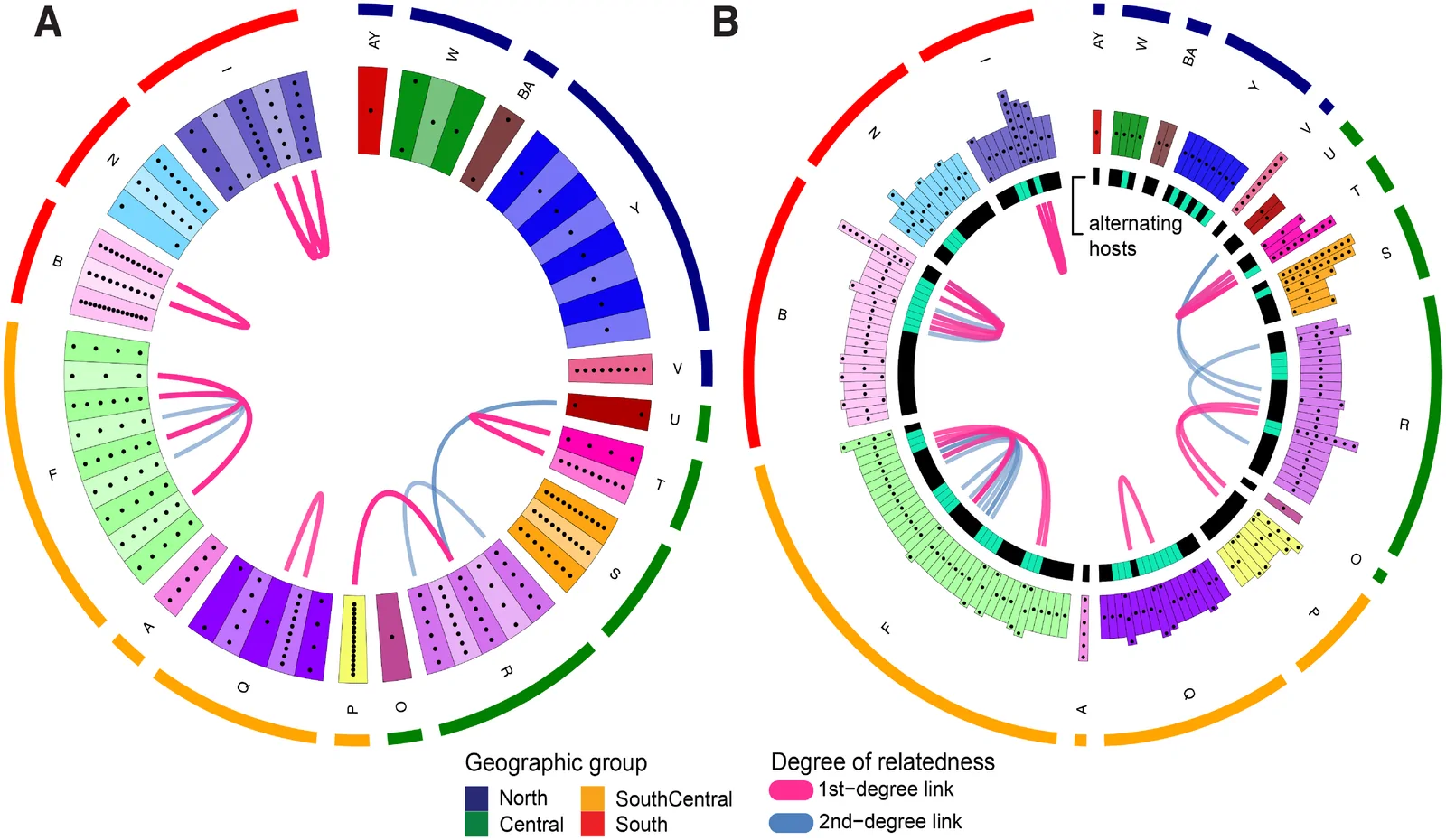
Host- and sib-cluster–level organization of close-kin relationships among *Schistosoma japonicum* miracidia in Sichuan Province, China. (A) Circos visualization of first- and second-degree relationships grouped by human host. Tiles represent hosts, dots denote sampled miracidia, and arcs indicate kinship (pink = first degree; blue = second degree). Close-kin links frequently connect multiple hosts within the same village, consistent with localized transmission involving closely related parasite lineages. Eight first-degree clusters and four second-degree host pairs were detected, with nearly all links connected within villages and only rare connections spanning villages. (B) Visualization at the sib-cluster level shows the distribution of genetically inferred sibling groups and variation in inferred worm burden. Inner bars represent sibling clusters with alternating host membership, and dots again denote individual miracidia. Hosts with higher worm burden more frequently participate in between-host kinship links, suggesting a greater contribution to local transmission networks. Outer colored arcs denote geographic clusters.

**Fig 6:**
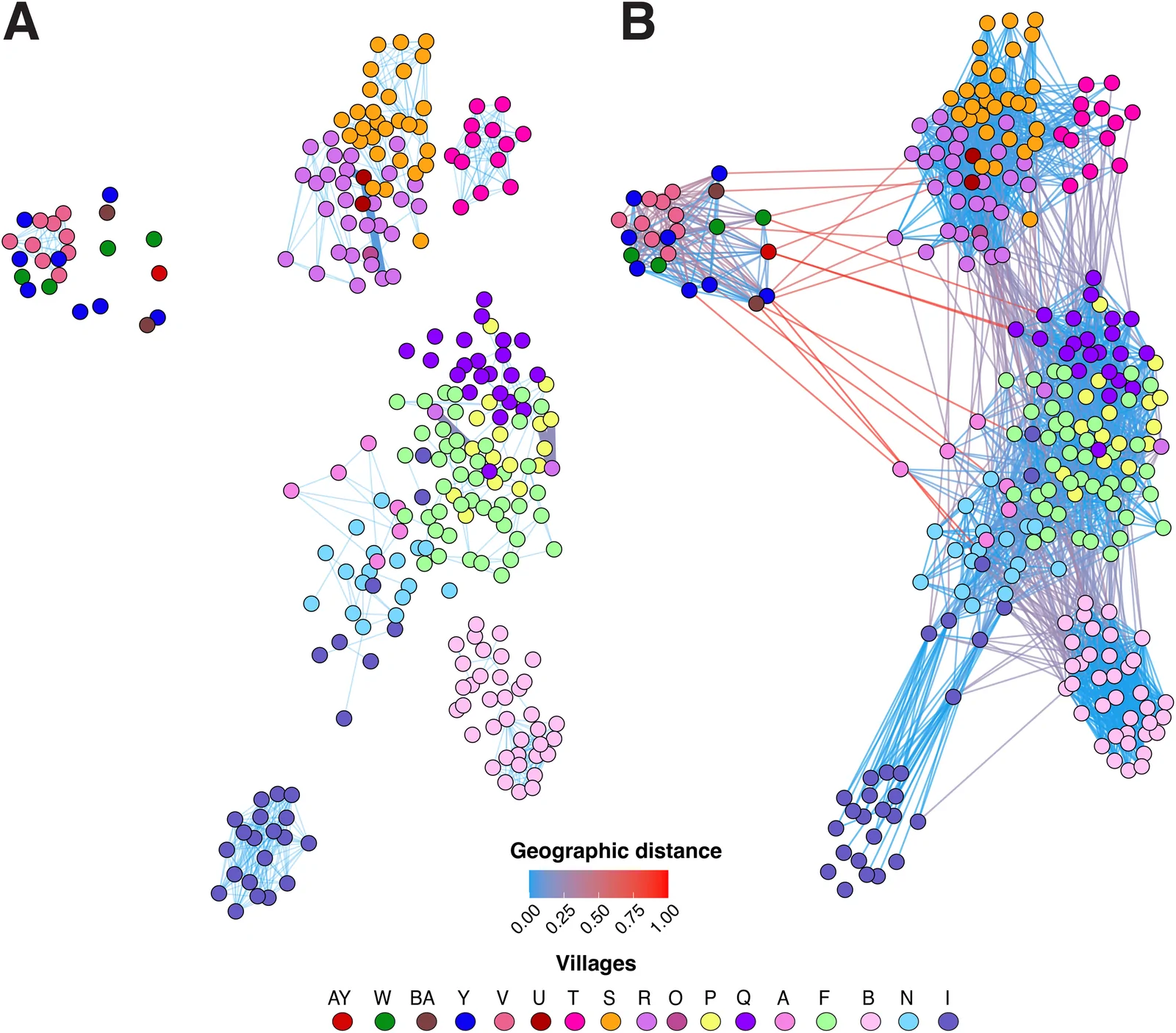
Network structure of close-kin and third-degree relationships among *S. japonicum* miracidia. Nodes represent individual miracidia and are colored by village of origin (north–south order shown in legend). Edges connect pairs of individuals inferred to be related with ≥95% posterior probability. Edge color reflects rescaled geographic distance between villages (blue = short distance; red = long distance). (A) First- and second-degree relationships (close kin) form largely village-contained clusters, with only rare cross-village links. Highlighted red connections indicate close-kin links between villages R–P and R–U. (B) Third-degree relationships reveal a substantially broader and more continuous spatial network, frequently connecting adjacent villages and extending across regional groupings (North, Central, Southcentral, South).

Although closely related parasites were predominantly found within villages, we identified rare but notable instances of first- and second-degree relationships spanning villages. At the host level (Fig 5A), visualization of ≥95% posterior first- and second-degree links shows that nearly all (96.6%) close-kin connections occur among hosts within the same village. Eight first-degree clusters and four second-degree host pairs were detected, with only two connections linking different villages. One notable exception involved a host from village R that shared a first-degree relationship with a host in village P and a second-degree relationship with a host in village U. Apart from this cluster, close-kin relatedness appears highly localized, indicating that recent direct transmission between villages was uncommon. Visualization at the sibling-cluster level (Fig 5B) provides additional resolution. Rather than grouping links by host, this organizes connections by genetically inferred sibling clusters within hosts, which approximate distinct reproducing worm pairs within hosts. This representation similarly reveals that a subset of sibling clusters contributes disproportionately to between-host genetic links. Of the 152 identified sibling clusters, 33 had a first- or second-degree relationship with a sibling cluster from another host within the village, and 7 had a first- or second-degree relationship with a sibling cluster from another village. To further resolve the spatial topology of close-kin connections, we visualized first- and second-degree relationships as an individual-level network (Fig 6A). Consistent with the host- and sib-cluster–level patterns, close-kin relationships form tightly clustered, village-contained components, with only rare cross-village links. Notably, the few between-village connections are restricted to specific village pairs (R–P and R–U), reinforcing the conclusion that recent transmission is highly localized and only occasionally spans village boundaries.

These network-based observations are consistent with patterns visible in the RAB heatmap introduced earlier (Fig 4). In addition to the prominent diagonal village-level blocks, the RAB heatmap reveals sparse but distinct off-diagonal hotspots of elevated relatedness connecting hosts from different villages. While first- and second-degree cross-village links are rare, moderate off-diagonal signals – corresponding primarily to second- and third-degree relationships – are more widespread, particularly within the central geographic cluster. This matrix-based view reinforces the conclusion that *S. japonicum* relatedness is not structured strictly by geographic distance, as several geographically separated villages share detectable genetic connections.

In contrast to the rarity of close-kin cross-village links, third-degree relationships reveal a broader and more continuous spatial network (Fig 6B). Numerous third-degree links connect hosts across villages, frequently uniting geographically adjacent villages and, in some cases, spanning larger regional groupings. While certain villages remain relatively self-contained (e.g., village B), most are part of multi-village networks of relatedness structured largely by the four geographic village groups (North, Central, Southcentral, and South). Importantly, this broader network mirrors the weak north–south differentiation observed in PCA and ADMIXTURE analyses, reinforcing the conclusion that *S. japonicum* populations across villages were not fully isolated. Together with the absence of recent demographic collapse, these results indicate that village-level transmission occurred recently (relative to our sampling) within a regionally connected schistosome population.

### Heterogeneity in genomically inferred worm burden

We used the sibling cluster assignments to infer the minimum number of reproducing adult worm pairs contributing to infection within a given host, providing a genomic estimate of the minimum worm burden. Each sibling cluster was assumed to represent a distinct reproducing worm pair; consequently, inferred worm burden should be interpreted as a conservative minimum estimate. Because sampling depth varied among hosts, all analyses explicitly accounted for differences in the number of sampled miracidia. Among the 23 hosts with five or more sampled miracidia, inferred minimum worm burden ranged from one to eleven sibling clusters (equivalent to 2–22 reproducing adult worms), with considerable heterogeneity observed among both hosts and villages (S6 Fig). Villages B and R exhibited consistently higher inferred worm burdens (B: mean = 7; R: mean = 4.6), whereas village I showed consistently lower burdens (mean = 2.6). Similarly, among the seven hosts with more than nine sampled miracidia, two hosts from village S each yielded 10–11 sampled miracidia derived from a single parental worm pair, whereas hosts from villages P, B, and R contained between 6 and 11 distinct sibling clusters (S7 Fig), illustrating clear differences in infection composition among hosts. To quantify heterogeneity in inferred worm burden, we fit a zero-truncated negative binomial distribution to sibling-cluster counts across all 53 hosts. The estimated dispersion parameter (k = 0.69; 95% CI: 0.14-3.40) falls well within the range reported for aggregated wildlife macroparasite populations (typically k < 1; [44]).

## Discussion

### Multi-scale genomic evidence of re-emergence in a near-elimination setting

In this retrospective genomic analysis of parasites collected immediately following documented re-emergence, we identified complementary genomic signatures that provide insight into the processes that enabled re-emergence despite decades of intensive control [47]. Demographic analyses indicated that the sampled parasites descended from a broadly cohesive regional population that retained substantial genetic diversity and showed no evidence of a recent decline in effective population size despite decades control efforts. At finer scales, patterns of relatedness among parasites sampled from humans revealed localized clusters of closely related parasites, consistent with infections from a limited number of infected snails. Although first- and second-degree relationships were largely observed within-villages, third-degree relationships indicated low-frequency genetic connectivity among villages. Together, these findings suggest that long-term demographic history and the recent genetic structure of sampled infections provide complementary perspectives on the processes underlying re-emergence. While the demographic signal indicates that the sampled parasites originated from a large and genetically diverse breeding population, the fine-scale relatedness patterns suggest that human infections present during re-emergence were already highly localized and dominated by relatively few parasite lineages. Below, we discuss how interpreting these distinct signals together provides a more complete picture of why re-emergence remained possible despite decades of successful control than either analysis alone.

### Demographic stability despite sustained control

Schistosomiasis elimination programs assume that sustained reductions in human prevalence will eventually drive schistosome populations below density-dependent transmission thresholds, leading to demographic collapse [14–16]. Our genomic results from Sichuan suggest a more complex relationship between declining human prevalence and *S. japonicum* population size. Despite decades of mass drug administration (MDA), snail control, and sanitation improvements [10,11], the sampled parasites retained substantial standing genetic diversity and showed no genomic evidence of a recent decline in effective population size. For example, standing genetic diversity in Sichuan *S. japonicum* miracidia (π ≈ 0.0015) was approximately half that reported for East African *S. mansoni* populations at much earlier stages of control (π ≈ 0.0033) [26], indicating that considerable genetic diversity persisted despite the prolonged history of control in Sichuan and across China. Importantly, however, demographic reconstructions describe the long-term ancestry of the sampled parasites rather than contemporary census population size or the locations and host species in which parasite reproduction occurred.

One explanation for the lack of demographic collapse is continued schistosome reproduction occurring in a broader host community. Because demographic reconstructions reflect the long-term ancestry of the sampled parasites rather than the locations or host species in which that reproduction occurred, our genomic data cannot identify which hosts maintained the breeding population represented by these parasites. However, *S. japonicum* infects multiple mammalian hosts, including agricultural animals such as bovines and pigs, and commensal species such as dogs, cats, and rodents [10,48–51]. Although relative contributions from these hosts may vary, evidence from eastern China suggests bovines likely play a major role in maintaining local schistosome reservoirs, and sustaining a major fraction of human transmission [51,52]. In multi-host systems, effective population size reflects reproduction across the broader host community rather than within humans alone. Thus, the persistence of a relatively large effective population size despite declining human prevalence is consistent with continued parasite reproduction occurring outside the sampled human population. We do not interpret these findings as evidence that control failed. Rather, they suggest that the measured reductions in reported human prevalence may reflect a reshaping of the broader transmission system, while allowing parasite effective population size to remain above maintenance levels. These findings have important implications for elimination strategies that rely primarily on human prevalence as an indicator of interruption.

These demographic interpretations should be considered in light of the limitations of demographic inference from genomic data. Power simulations confirmed that both Stairway Plots and SMC++ reliably detect bottlenecks of the timing and severity relevant to our hypothesis, supporting our conclusion that no recent demographic decline is evident. However, the same simulations showed that both methods become less precise at shallow time depths, with Stairway Plots tending to overestimate recent N_e_ and SMC++ exhibiting greater replicate-to-replicate variability. Population subdivision and the hierarchical structure of our sampling, inherent to a multi-host, multi-village system, may also influence the precise magnitude and timing of inferred historical N_e_ [46]. These considerations warrant caution when interpreting the fine details of the reconstructed demographic trajectory. Importantly, however, demographic reconstructions describe the long-term ancestry of the sampled parasites rather than contemporary census population size or the locations and host species in which parasite reproduction occurred. Consequently, these analyses constrain the demographic history represented by the sampled parasites but do not identify the ecological or epidemiological processes responsible for maintaining that diversity.

### Localized transmission and clonal amplification within villages

Whereas demographic analyses describe the long-term ancestry of the sampled parasites, patterns of relatedness, inbreeding, and heterozygosity provide insight into the more recent genetic structure of infections present during re-emergence. These complementary genomic perspectives help explain why regional demographic stability and highly localized genetic structure are not contradictory, but instead reflect different biological and temporal scales of the parasite population.

Viewed together, these finer-scale genomic patterns suggest that control efforts substantially altered the organization of parasite populations without producing regional demographic collapse. The relatively high frequency of full- and half-sibling relationships recovered among miracidia sampled from different human hosts are difficult to reconcile with large numbers of independently infected snails, but are readily explained by repeated infections from clonally related cercariae released by a relatively small number of infected snails. Intensive snail control is expected to reduce the number of infected snails contributing to human infection, thereby narrowing the pool of parasite lineages available to seed local infections. Our results are remarkably consistent with this expectation, suggesting that relatively few infected snails contributed disproportionately to the sampled human infections while still maintaining localized transmission.

This mosaic structure of localized bottlenecks nested within a stable metapopulation is consistent with host–parasite metapopulation theory [21,53]. In such systems, patchy local extirpation and recolonization can occur without regional or global elimination. Such recolonization may be facilitated by the long lifespan of adult schistosomes in mammalian hosts [6], together with occasional movement of infected humans or other mammalian hosts that can re-establish local transmission foci. Together, these observations suggest that control substantially narrowed – but did not completely sever – the ecological connections necessary to sustain parasite reproduction. The resulting mosaic of localized persistence embedded within a regionally connected parasite population provides a plausible genomic framework for understanding why re-emergence remained possible despite decades of successful control.

### Limited dispersal but evidence of regional connectivity

Although first- and second-degree relationships were largely confined to individual villages, third-degree relationships frequently spanned geographic clusters, indicating that *S. japonicum* lineages were not completely isolated over longer timescales. These more distant relationships are consistent with infrequent genetic exchange mediated through multiple, non-exclusive pathways, including hydrologic connectivity among snail habitats, dispersal of infected snails or cercariae through connected waterways, and movement of infected humans or other mammalian hosts [20,23,54–56]. The spatial distribution of these third-degree relationships closely mirrors the weak north–south genetic gradient identified by PCA and ADMIXTURE analyses, reinforcing the conclusion that villages remained components of a broader regional population.

Importantly, our results suggest that only modest levels of connectivity may be sufficient to maintain the regional breeding population despite extensive fragmentation of local transmission. While localized transmission appears to have been sustained by relatively few infected snails within individual villages, infrequent genetic exchange among villages likely prevented complete genetic isolation and buffered the regional parasite population against local extinction. Although we cannot distinguish among the aforementioned hydrological or human/mammalian movement pathways with genomic data alone, they collectively provide biologically plausible mechanisms for maintaining regional connectivity despite increasingly localized transmission. This hierarchical organization is consistent with metapopulation theory [22,53], in which weak but persistent connectivity among localized transmission foci can maintain regional persistence despite frequent local bottlenecks. From a control perspective, these findings suggest that reducing transmission below apparent epidemiological thresholds may not be sufficient if even infrequent dispersal continues to reconnect local parasite populations and replenish regional genetic diversity [20].

### Variation in worm burden reflects differences in exposure across villages

Our analyses suggest that variation in worm burden may further shape fine-scale patterns of local infection. Using sibling clusters as proxies for the minimum number of distinct reproducing worm pairs per host, we observed measurable variation in minimum worm burden across hosts and villages. Some hosts harbored parasites derived from a single adult worm pair despite multiple sampled miracidia, whereas others contained numerous genetically distinct sibling clusters, indicating multiple concurrent worm pairs and (in some cases over 20 inferred reproducing adult worms) within a single host. These estimates should be considered conservative lower bounds because incomplete sampling inevitably underestimates the total number of reproducing worm pairs present within each host.

A minority of hosts appeared to harbor substantially larger numbers of inferred reproducing worm pairs than most others, consistent with well-documented heterogeneity in host exposure and the characteristic aggregation of macroparasite infections [14,18,57]. To formally evaluate this pattern, we fit a zero-truncated negative binomial distribution to inferred worm burdens across all 53 hosts, yielding a dispersion parameter of k = 0.69 (95% CI: 0.14–3.40), well within the range typically reported for macroparasite populations [44]. Thus, despite decades of sustained control and the extensive restructuring of schistosome populations revealed by our analyses, the overall degree of worm burden aggregation remained broadly consistent with one of the most pervasive ecological patterns observed among macroparasites. Rather than indicating unusually concentrated transmission, these findings suggest that the ecological processes generating heterogeneous host exposure remain largely intact even in this near-elimination setting.

Genomic pedigree data, however, provide an additional dimension that conventional estimates of worm burden cannot. Hosts inferred to harbor larger numbers of genetically distinct reproducing worm pairs also tended to share close pedigree relationships with parasites recovered from a greater number of other hosts, suggesting that variation in parasite burden may also influence how parasite lineages are distributed within local transmission networks. Although these observations do not demonstrate disproportionate transmission directly, they are consistent with the broader principle that heterogeneity among hosts can influence parasite persistence and transmission dynamics [18,55]. Viewed alongside the strong pedigree structure observed within villages, these findings suggest that localized transmission remained heterogeneous at multiple biological levels: relatively few infected snails appear to have generated many related infections, while some hosts harbored substantially greater numbers of genetically distinct reproducing worm pairs than others, consistent with marked heterogeneity in parasite exposure and or host susceptibility [58].

Extending genomic pedigree analyses to estimate the minimum number of unique reproducing worm pairs at the village level further illustrates how genomic metrics can inform elimination efforts. Villages S and B exhibited comparable infection prevalence and sampling depth, yet differed by nearly four-fold in the inferred number of worm clusters, demonstrating that prevalence alone can obscure important differences in parasite exposure and the diversity of reproducing parasite lineages within communities. Integrating genomic estimates of worm burden with relatedness-based transmission mapping may therefore provide a more informative framework for prioritizing surveillance and targeted interventions during the final stages of schistosomiasis elimination.

### Evidence from subsequent genomic surveillance

An important question raised by our analyses is how this system responded to continued control following the 2007 re-emergence. While our study provides a genomic snapshot immediately after resurgence, subsequent genomic studies from the same endemic region offer an opportunity to evaluate how these transmission patterns evolved under continued intervention. In a prior study, Shortt et al. [39] analyzed *S. japonicum* collected between 2008 and 2016 using an independent reduced-representation genomic dataset to estimate relatedness and transmission networks among human hosts. Despite differences in sampling period, genomic approach, and marker density, their results provide an independent longitudinal perspective that closely complements the genomic framework inferred here. Compared to our 2007 samples, they found that schistosomes in this region during these later periods became more strongly structured among villages, indicating that continued control progressively disrupted the broader regional connectivity evident immediately following re-emergence. However, parasites within villages remained highly related and infections were frequently derived from a limited number of local transmission sources, suggesting that localized transmission persisted despite reductions in regional dispersal [39].

Together, these studies suggest that the disruption of schistosome transmission under sustained control proceeded hierarchically across spatial scales in Sichuan. Continued control appears to have progressively reduced regional connectivity among transmission foci while leaving localized village-level transmission networks comparatively intact. This progression closely matches the framework developed here, in which weak regional connectivity can be disrupted while small, locally sustained transmission networks remain sufficient to maintain parasite persistence. From a control perspective, these findings suggest that breaking regional transmission alone may be insufficient for durable elimination if persistent local transmission foci remain. As elimination programs advance, increasing emphasis may therefore need to be placed on identifying and eliminating these remaining village-scale reservoirs through integrated surveillance of human infections, non-human reservoir hosts, and local snail populations.

### Environmental persistence and incomplete disruption of transmission pathways

The localized clustering of closely related parasites within villages, together with rare cross-village links, implicates environmental persistence as a key component of continued transmission. *S. japonicum* depends on *Oncomelania* snails, whose distribution is shaped by hydrology, agriculture, and landscape structure [23, 54]. Water networks that sustain *Oncomelania* habitats and concentrate human and livestock activity likely serve as primary conduits of schistosome movement between neighboring transmission sites [20]. Even under sustained snail control, small residual habitats can maintain infected snail populations capable of generating focal outbreaks through clonal cercarial amplification. Our findings do not imply widespread uncontrolled transmission. Rather, elimination efforts appear to have fragmented transmission into localized foci without fully severing ecological and spatial pathways necessary for long-term persistence. In such contexts, re-emergence may arise from expansion of environmentally buffered transmission units within a weakly connected regional network. Targeting residual environmental linkages may therefore be as important as continued chemotherapy in late-stage elimination. Integrating genomic surveillance with fine-scale ecological mapping could help identify persistent transmission corridors that prevalence monitoring alone may miss.

### Study limitations and future directions

Our analyses provide a retrospective genomic snapshot of *S. japonicum* human infections immediately following documented re-emergence in Sichuan, but several limitations identify important directions for future work. Most notably, our sampling was restricted to human-derived miracidia. Consequently, although our demographic and pedigree analyses strongly support continued regional connectivity and localized transmission, they cannot determine the relative contributions of infected humans, non-human mammalian reservoirs, infected *Oncomelania* snails, or hydrologically connected waterways to parasite movement across the landscape. Likewise, demographic analyses describe the long-term ancestry of sampled parasites rather than the specific host species or ecological settings in which parasite reproduction occurred. Population genomic sampling of parasites from humans, non-human mammalian reservoirs, and infected *Oncomelania* snails, integrated with ecological and hydrological data, will be essential for resolving these remaining transmission pathways and identifying persistent transmission corridors in near-elimination settings [7,9,24,52,54].

More broadly, recent perspectives have emphasized that demographic inference from genomic data should be interpreted cautiously, particularly when reconstructing historical population size from complex natural populations [46]. In *Schistosoma*, these challenges are compounded by features of the parasite’s biology, including its multi-host life cycle, clonal amplification within intermediate snail hosts, repeated transmission bottlenecks, and hierarchical population structure resulting from parasites nested within hosts and hosts nested within villages. Together, these processes may complicate the assumptions underlying commonly used demographic models and reduce the precision with which historical changes in effective population size can be inferred, particularly in near-elimination settings where transmission becomes increasingly heterogeneous. Consequently, demographic estimates should be interpreted as approximations of long-term evolutionary history rather than precise measures of contemporary census size or transmission intensity. Importantly, however, the principal biological conclusions of this study do not depend on any single demographic estimate. Rather, they emerge from the convergence of multiple independent genomic analyses – including demographic reconstruction, pedigree inference, relatedness, inbreeding, spatial population structure, and worm burden estimation – that consistently support regional persistence coupled with sustained localized transmission across multiple biological scales.

Our sampling depth also varied among villages and hosts, with some villages represented by a single infected host and miracidia per host ranging from one to sixteen (S2 Table). Consequently, estimates of village-level relatedness and minimum worm burden should be interpreted as conservative lower bounds, and genomic patterns at sparsely sampled locations may not fully capture local infection networks. These limitations are inherent to retrospective analyses of historical collections and cannot be readily overcome for the present dataset. However, they highlight important considerations for future genomic surveillance efforts. More balanced sampling across hosts and villages, broader geographic coverage, and repeated longitudinal sampling would substantially improve the resolution with which multi-scale transmission networks can be reconstructed and their contraction across spatial scales monitored as elimination programs progress.

### Implications for elimination strategy and genomic surveillance

China’s schistosomiasis control program remains among the most successful globally, achieving dramatic reductions in morbidity and human infection prevalence over the past several decades [8,11]. Our findings do not diminish these remarkable achievements. Rather, they illustrate that in environmentally mediated, multi-host parasite systems such as *S. japonicum*, transmission may become increasingly fragmented without a proportional loss of regional genetic diversity or complete disruption of transmission networks. Consequently, conditions that permit re-emergence may persist long after human prevalence has declined to very low levels.

Recent studies have increasingly demonstrated the value of population genomic approaches for understanding schistosome transmission, connectivity, parasite movement, and persistence under sustained control [20,25,26,59]. Collectively, these studies illustrate how genomic data can complement traditional epidemiological surveillance by revealing aspects of parasite population structure that are otherwise difficult to observe directly. Our study builds upon these advances by integrating demographic reconstruction, pedigree inference, relatedness, spatial population structure, and minimum worm burden estimation within a single analytical framework. Together, these complementary analyses provide a multi-scale perspective on parasite persistence spanning evolutionary history, regional connectivity, village-level transmission, and host-level infection dynamics, illustrating how genomic data can simultaneously inform multiple components of the transmission process.

Beyond reconstructing transmission networks, genomic surveillance also offers opportunities to monitor the evolutionary responses of parasite populations to sustained control. As genomic resources continue to expand, these approaches may facilitate the detection and monitoring of loci associated with host adaptation, transmission dynamics, and the emergence or spread of reduced drug susceptibility before widespread treatment failure becomes clinically apparent [20,26,46]. Viewed together, these developments suggest that population genomics is becoming an increasingly valuable complement to traditional epidemiological surveillance. By integrating information across evolutionary, demographic, and epidemiological timescales, genomic approaches have the potential to characterize transmission networks, monitor parasite adaptation, and ultimately support more targeted and durable elimination strategies.

## Supporting information

S1 Table Infection prevalence and mean village infection intensity. The estimated prevalence of infections and mean village infection intensity in 2007 for each village included in this study. Prevalence and infection intensity estimates are based on methods described in Carlton et al [17].
(XLSX)

S2 Table Village-level sampling structure of the 2007 Sichuan miracidia dataset. Number of human hosts and miracidia sampled per village, with the range of samples collected per host, across all 17 villages included in this study (53 hosts, 270 miracidia total). Villages are identified by the same letter codes used throughout the main text and figures.
(XLSX)

S1 Text Whole genome amplification protocol. Modified GenomiPhi V3 whole-genome amplification protocol for DNA extracted from individual FTA card punches.
(DOCX)

S2 Text Illumina library preparation protocol and sequencing. Nextera DNA Flex library preparation protocol for sequencing libraries generated from individual WGA products. Library pooling strategy and NovaSeq 6000 sequencing parameters for the 270-sample miracidia dataset.
(DOCX)

S1 Fig Sequencing quality metrics across 270 *S. japonicum* miracidia samples. Top panel shows the distribution of the percentage of reads mapped to the *S. japonicum* reference genome. Most samples exhibited high mapping rates (>95%), indicating strong enrichment for parasite DNA and minimal host contamination. Bottom panel shows the distribution of mean genome-wide sequencing coverage per sample. Coverage was generally centered between ~20× and 35 × , with a small number of higher-coverage outliers. Together, these results demonstrate consistently high-quality whole-genome sequencing suitable for downstream population genomic and relatedness analyses.
(TIF)

S2 Fig Population structure inferred using ADMIXTURE. Cross-validation (CV) error values across K = 1–12 indicate minimal support for additional population structure beyond K = 1. Bar plots show individual ancestry proportions for K = 2 and K = 3 based on a 10-kb thinned genome-wide SNP dataset. Each vertical bar represents a single miracidium, with colors indicating inferred ancestry components. Individuals are ordered by village following a north–south geographic gradient, with village identity indicated by the color bar below. At K = 2, parasites form two broadly distributed ancestry components with limited geographic structuring. At K = 3, additional subdivision emerges, though patterns remain diffuse and do not correspond strongly to geographic groupings, consistent with weak population structure and ongoing connectivity across villages.
(TIF)

S3 Fig Bootstrap-collapsed neighbor-joining tree of 270 Sichuan 2007 isolates, rooted with two outgroups and grouped by village. Neighbor-joining tree built from pairwise genetic distances using a 10-kb thinned SNP set (to avoid inflating support from linked sites), with 272 tips total including two outgroup samples. Branches with <85% bootstrap support (10,000 replicates) were collapsed into polytomies. Tips are colored by village and reordered into contiguous village blocks by geographic cluster (North, Central, South-Central, South) for visual clarity; this preserves all resolved branching structure but overrides strict tip order for a small number of tips.
(TIF)

S4 Fig Power simulations evaluating SMC++ and Stairway Plot sensitivity to recent effective population size decline. Ne trajectories inferred from coalescent power simulations under a constant-Ne null (A) and simulated bottlenecks of 2× (B), 10× (C), and 50× (D) severity, with onset 60 generations before sampling to approximate the onset of mass drug administration in 1977. For each scenario, simulated genotypes were analyzed using both SMC++ (median across 5 replicates, corrected time-point configuration, timepoints = 10) and Stairway Plot (median across 20 replicates), alongside the true simulated Ne (black, dashed). Both methods recover the constant-Ne null and detect the 10× and 50 × bottlenecks, while showing reduced sensitivity and greater deviation from truth at 2 × severity, consistent with known limitations of coalescent-based Ne inference at shallow time depths.
(TIF)

S5 Fig Concordance between RAB and RAS relatedness metrics and pedigree-based classifications. (A) Scatterplot of pairwise relatedness estimates comparing RAB (x-axis) and RAS (y-axis) across all pairs of *S. japonicum* miracidia. The dashed line indicates the fitted linear relationship (y = 0.592x), with R² = 0.364, demonstrating a strong positive correlation between the two metrics. (B) Same comparison colored by Sequoia-inferred top relationship class (FS = full siblings; HA = half siblings; U = unrelated). Full- and half-sibling pairs cluster at higher RAB and RAS values, consistent with increasing genetic relatedness. (C) Same comparison colored by COLONY full-sibling assignments (probability ≥ 1), showing substantial concordance between COLONY full-sibling calls and elevated RAB/RAS values. Together, these results demonstrate agreement between likelihood-based relatedness metrics and pedigree reconstruction approaches.
(TIF)

S6 Fig Village-level network of third-degree genetic relationships across Sichuan. Circular network (circos) plot summarizing the number and degree of pairwise genetic relationships among villages. Each outer segment represents a village, ordered geographically, with the outermost colored band indicating geographic group (North, Central, Southcentral, South). Chords connecting villages represent inferred relatedness links between miracidia sampled from different villages, colored by degree of relatedness (1st-degree, 2nd-degree, 3rd-degree). Link width is proportional to the number of inferred relationships between village pairs. First- and second-degree connections are rare and largely localized, whereas third-degree links are widespread and form a dense, regionally connected network spanning multiple geographic groups.
(TIF)

S7 Fig Distribution of parasite samples and relatedness within hosts across villages. Bars show the total number of *Schistosoma japonicum* miracidia sampled from each host, while black points indicate the number of observed related pairs (RAB ≥ 0.4) detected among parasites within that host. Hosts are ordered geographically from north to south by village to highlight regional patterns in parasite sampling and relatedness structure. Bar colors indicate village of origin, and the colored strip along the bottom denotes broader geographic clusters (North, Central, SouthCentral, and South). Variation in both sample counts and the number of related parasite pairs among hosts reflects heterogeneity in inferred worm burdens and transmission structure across the study region.
(TIF)

S8 Fig Bootstrap mean effective population size trajectories for *Schistosoma japonicum* in Sichuan Province. (A) Demographic history inferred using the sequential Markov coalescent approach (SMC++), showing the bootstrap mean trajectory across n = 10 replicates. (B) Historical effective population size (Ne) inferred from site-frequency spectra using Stairway Plot, showing the bootstrap mean trajectory across n = 200 replicates. Both panels show effective population size (Ne) as a function of time before present (years, assuming a 0.5-year generation time). Trajectories are consistent with the median-based estimates presented in the main text (Fig 2B and 2C), indicating a deep historical decline without evidence of a pronounced recent reduction coincident with modern schistosomiasis control.
(TIF)
