# Supplementary material for: Population genomics reveals multi-scale mechanisms sustaining schistosomiasis re-emergence in a near-elimination setting
Source: PLoS Negl Trop Dis. 2026 Sep 8;20(9):e0014202. doi: 10.1371/journal.pntd.0014202 (PMC13581208; doi:10.1371/journal.pntd.0014202)
Supplement: Supplementary file 1 [file pntd.0014202.s003.docx]

## S1 Text: Whole Genome Amplification Protocol

Whole genome amplification (WGA) was performed using the Illustra Ready-To-Go GenomiPhi V3 DNA Amplification Kit (GE Healthcare; cat. 25-6601-96), following a modified version of the General Protocol (Section 5.2). The protocol below was developed by Jonathan Shortt and incorporates specific deviations from the manufacturer’s standard instructions as described.

**Sample preparation and FTA card punch washing**

Punch tubes were organized by box order and miracidium ID. Each 2 mm FTA card punch was washed sequentially with 150 μL FTA Purification Reagent (3 × 5 min) followed by 150 μL 1× TE buffer (2 × 5 min). Punches were then air-dried for 1 hour in a fume hood under a kimwipe to prevent contamination, before being transferred individually to labeled 0.2 mL PCR tubes using a 1000 μL pipette tip.

**DNA denaturation**

20 μL of 1× denaturation buffer was added to each punch tube. Note: the kit-supplied denaturation buffer is provided at 2× concentration and was diluted to 1× with molecular biology-grade water prior to use. DNA was denatured in a thermal cycler at 95°C for 3 minutes, followed by a hold at 4°C.

**Whole genome amplification**

GenomiPhi V3 reaction cakes were reconstituted with denatured template DNA as follows. Tubes containing denatured template and reaction cakes were placed on ice. Using a multichannel pipette, 20 μL of denatured template was transferred from each punch tube to the corresponding cake tube and incubated on ice for 10 minutes. The liquid was then returned to the original punch tube using a multichannel pipette. Amplification was performed in a thermal cycler: 90 minutes at 30°C (amplification), followed by 10 minutes at 65°C (heat inactivation), and a hold at 4°C.

**Post-amplification quantification and storage**

Amplified DNA was quantified using a Qubit fluorometer. Samples yielding ≥20 ng total DNA were selected for downstream library preparation. Punches were returned to their original labeled Eppendorf tubes, parafilmed, and stored at −20°C.
