# Supplementary material for: Population genomics reveals multi-scale mechanisms sustaining schistosomiasis re-emergence in a near-elimination setting
Source: PLoS Negl Trop Dis. 2026 Sep 8;20(9):e0014202. doi: 10.1371/journal.pntd.0014202 (PMC13581208; doi:10.1371/journal.pntd.0014202)
Supplement: Supplementary file 2 [file pntd.0014202.s004.docx]

## S2 Text: llumina Library Preparation Protocol

Sequencing libraries were prepared from individual WGA products using the Illumina Nextera DNA Flex Library Preparation Kit, following the manufacturer’s instructions with the modifications noted below. Input DNA was normalized to a target of 100–500 ng (optimal target: 250–300 ng) and diluted in molecular biology-grade water as needed.

**Tagmentation**

2–30 μL of normalized DNA was added to each well of a 96-well PCR plate and brought to 30 μL total volume with molecular biology-grade water. Bead-Linked Transposomes (BLT) were vortexed vigorously until fully resuspended. A tagmentation master mix was prepared by combining 11 μL BLT and 11 μL Tagmentation Buffer 1 (TB1) per sample and vortexed thoroughly. 20 μL of tagmentation master mix was added to each sample well and pipetted up and down 10 times to mix. The plate was sealed and run on a thermal cycler using the TAG program: 55°C for 15 minutes (preheated lid at 100°C, 50 μL reaction volume), followed by a hold at 10°C.

**Post-tagmentation cleanup**

10 μL Tagment Stop Buffer (TSB) was added to each well and pipetted to resuspend beads. The plate was run on the PTC thermal cycler program: 37°C for 15 minutes (preheated lid at 100°C, 60 μL reaction volume), followed by a hold at 10°C. The plate was then placed on a magnetic stand until the solution cleared (∼3 minutes), and the supernatant was discarded. Beads were washed twice with 100 μL Tagment Wash Buffer (TWB), with full resuspension and magnetic clearing between washes. Following the final wash, beads were resuspended in 100 μL TWB and kept on the magnetic stand until the PCR amplification step.

**PCR amplification and indexing**

A PCR master mix was prepared by combining 22 μL Enhanced PCR Mix (EPM) and 22 μL molecular biology-grade water per sample. The supernatant was removed from beads on the magnetic stand, and 40 μL PCR master mix was added directly to each well. Unique dual indexes were added to each sample and mixed by pipetting. PCR was performed using the BLT PCR program: 68°C for 3 minutes, 98°C for 3 minutes, followed by 7 cycles of 98°C for 45 seconds, 62°C for 30 seconds, and 68°C for 2 minutes, with a final extension of 68°C for 1 minute and hold at 10°C. Libraries could be stored at 4°C for up to 3 days at this stage.

**Library cleanup**

Libraries were cleaned up using Illumina Purification Beads (IPB) and Resuspension Buffer (RSB). The plate was centrifuged at 280 × g for 1 minute, placed on a magnetic stand for 5 minutes, and 45 μL of supernatant was transferred to a deep-well midi plate. IPB-based size selection was performed using a two-step bead cleanup: first, 40 μL water and 45 μL SPB were added to remove large fragments; supernatant was then transferred to a new midi plate containing 15 μL undiluted IPB to capture the final library. After a 5-minute room temperature incubation and magnetic clearing, beads were washed twice with 200 μL freshly prepared 80% ethanol, air-dried for 5 minutes on the magnet, and eluted in 32 μL RSB. Final eluted volume was 30 μL.

## Library Pooling and Sequencing

Individual indexed libraries were quantified and assessed for fragment size distribution prior to pooling. Libraries were pooled in multiplexed sets of up to 96 samples, with each library contributing equally to the pool based on molar concentration. The final pooled library was prepared at 4 nM in 50 μL and submitted to the North Texas Genome Center for sequencing.

Sequencing was performed on an Illumina NovaSeq 6000 instrument using an S4 flow cell with 150 bp paired-end reads. Sequencing depth targeted approximately 20× genome coverage per miracidium. Demultiplexing was performed using standard Illumina bcl2fastq software with default settings.
